# Detection of Novel Amplicons in Prostate Cancer by Comprehensive Genomic Profiling of Prostate Cancer Cell Lines Using Oligonucleotide-Based ArrayCGH

**DOI:** 10.1371/journal.pone.0000769

**Published:** 2007-08-22

**Authors:** Joern Kamradt, Volker Jung, Kerstin Wahrheit, Laura Tolosi, Joerg Rahnenfuehrer, Martin Schilling, Robert Walker, Sean Davis, Michael Stoeckle, Paul Meltzer, Bernd Wullich

**Affiliations:** 1 Department of Urology and Pediatric Urology, University of Saarland, Homburg/Saar, Germany; 2 Department of Computational Biology and Applied Algorithmics, Max-Planck Institute for Informatics, Saarbrücken, Germany; 3 Statistische Methoden in der Genetik und Chemometrie Fachbereich Statistik, Universität Dortmund, Dortmund, Germany; 4 Department of General, Visceral, Vascular and Pediatric Surgery, University of the Saarland, Homburg/Saar, Germany; 5 Genetics Branch, National Cancer Institute, National Institutes of Health, Bethesda, Maryland, United States of America; Lehigh University, United States of America

## Abstract

**Background:**

The purpose of this study was to prove the feasibility of a longmer oligonucleotide microarray platform to profile gene copy number alterations in prostate cancer cell lines and to quickly indicate novel candidate genes, which may play a role in carcinogenesis.

**Methods/Results and Findings:**

Genome-wide screening for regions of genetic gains and losses on nine prostate cancer cell lines (PC3, DU145, LNCaP, CWR22, and derived sublines) was carried out using comparative genomic hybridization on a 35,000 feature oligonucleotide microarray (arrayCGH). Compared to conventional chromosomal CGH, more deletions and small regions of gains, particularly in pericentromeric regions and regions next to the telomeres, were detected. As validation of the high-resolution of arrayCGH we further analyzed a small amplicon of 1.7 MB at 9p13.3, which was found in CWR22 and CWR22-Rv1. Increased copy number was confirmed by fluorescence *in situ* hybridization using the BAC clone RP11-165H19 from the amplified region comprising the two genes interleukin 11 receptor alpha (*IL11-RA*) and dynactin 3 (*DCTN3*). Using quantitative real time PCR (qPCR) we could demonstrate that *IL11-RA* is the gene with the highest copy number gain in the cell lines compared to *DCTN3* suggesting *IL11-RA* to be the amplification target. Screening of 20 primary prostate carcinomas by qPCR revealed an *IL11-RA* copy number gain in 75% of the tumors analyzed. Gain of *DCTN3* was only found in two cases together with a gain of *IL11-RA*.

**Conclusions/Significance:**

ArrayCGH using longmer oligonucleotide microarrays is feasible for high-resolution analysis of chomosomal imbalances. Characterization of a small gained region at 9p13.3 in prostate cancer cell lines and primary prostate cancer samples by fluorescence *in situ* hybridization and quantitative PCR has revealed interleukin 11 receptor alpha gene as a candidate target of amplification with an amplification frequency of 75% in prostate carcinomas. Frequent amplification of *IL11-RA* in prostate cancer is a potential mechanism of *IL11-RA* overexpression in this tumor type.

## Introduction

Genetic alterations are believed to be key events in the development of most tumors, including prostate cancer [1]. Tumor progression seems to depend on the successive acquisition of chromosomal aberrations leading to gains or losses of part of the tumor cell genome. Characterization of these genomic abnormalities in prostate cancer may therefore help to understand the molecular pathogenesis and may unveil genetic markers of progression.

Since its first description by Kallioniemi et al. (1992) [2] chromosomal comparative genomic hybridization (cCGH) has become the most frequently used technique to detect DNA copy number changes in tumor genomes. We and others have analyzed the genome of prostate cancer cell lines and primary prostate cancer samples with this technique [3]–[5]. Fluorescence *in situ* DNA hybridization (FISH) and quantitative real time PCR have been demonstrated to be valuable tools for target gene discovery within identified chromosomal regions of gain, e.g. the *TLOC1/SEC62* gene at 3q26.2 in prostate cancer [6]. Applying advanced bioinformatic models on cCGH data demonstrated that the patterns of chromosomal aberrations contain valuable prognostic information of a tumor [7].

Because of the relative low spatial resolution (∼20MB) of cCGH and its inaccuracy in centromeric as well as telomeric regions this technique is neither able to adequately detect small regions of gains or loses nor genomic alterations next to the centromere or telomere. Also, for target gene identification in gained regions as found by cCGH, fine-mapping with techniques like FISH is laborious and time-consuming.

Compared to cCGH, microarray-based CGH, referred to as array CGH (aCGH) or matrix CGH [8]–[9], has a roughly 1.000-fold higher resolution (or even higher) and allows analysis of chromosomal regions close to the centromere and telomere. Different approaches of aCGH have been followed over the years. Several groups utilized genomic BAC arrays [10] whereas others have chosen cDNA or oligonucleotide arrays that were originally designed for expression analysis [9], [11]. Arrays designed for gene expression are advantageous for direct comparison of genomic alterations and gene expression on the same platform. Several studies have demonstrated that this approach shows a significant association between gene copy number and expression level [12], [13]. Lately, use of oligonucleotide arrays specifically for aCGH designed longer was reported [14] and is now commercially available as an aCGH platform. For the aCGH analyses of prostate cancer cell lines as well as clinical specimens either BAC or cDNA arrays have been utilized [12], [13], [15]–[20].

Here we present the first study utilizing a 35,000 feature 70-mer oligonucleotide array, originally designed for expression analysis, for detailed genomic characterization of nine prostate cancer cell lines. Resulting aCGH profiles are compared to cCGH results. The occurrence of a newly detected small amplicon in the pericentromeric 9p13.3 subband in various cell lines is validated by FISH and quantitative real time PCR and is also confirmed in primary prostate cancer samples.

## Material and Methods

### Tumor cell lines and DNA isolation

The human prostate cancer cell lines DU145, PC3, LNCaP, CWR22 and CWR22-Rv1 were obtained from American Type Cell Culture Collection (ATCC, Rockville, MD, USA) and cultured according to the protocols recommended by the ATCC. From PC3 and DU145, two different branches were available, one held in the laboratory of the Cancer Genetic Branch, NHGRI, NIH (PC3*_NIH_*, DU145*_NIH_*), the other in the urological laboratory in Homburg (PC3*_HOM_*, DU145*_HOM_*). PC3-N, PC-125-1L, and DU145-MN1 were established as previously described [21], [22]. LNCaP-CN4-2 was kindly provided by Z. Culig, Department of Urology, Medical University, Innsbruck, Austria.

### Primary prostate cancer samples

Quantitative gene copy number measurements were done on 20 primary prostate adenocarcinoma samples, which were obtained after radical prostatectomy from previously untreated prostate cancer patients. Following prostatectomy, the specimens were dissected by a pathologist, snap frozen, and stored at −80°C. Only samples containing >50% tumor cells were included in the study.

### DNA isolation and quantification

DNA was isolated using the QiAmp DNA isolation kit (Qiagen, Hilden, Germany) and subsequently quantified by a fluorometric assay (Quant-iT Pico Green dsDNA Kit, Invitrogen, Karlsruhe, Germany). Fluorescence was measured using a TECAN (Salzburg, Austria) SpectrafluorPLUS microplate fluorescence reader with an excitation wavelength of 485 nm and an emission wavelength of 535 nm. DNA concentrations were calculated from a standard curve of double-stranded control DNA provided with the kit that was measured in triplicate at the concentrations 30, 3, 0.3, and 0.03 ng/µl (measured by fluorometry).

### Whole genome amplification and purification

A volume of 2.5 µl out of 4 ng/µl dilutions from cell lines and control DNA was used as starting material for the amplification. The phi29-amplification was carried out according to the Repli-G kit manufacturer's instructions (Qiagen) using an incubation time of 16 h. Repli-G reactions were checked by a real time based intra *Alu*-PCR assay. Additionally DNA concentration was fluorimetrically quantified with Quant-iT Pico Green dsDNA Kit (Invitrogen) and ranged between 10–30 μg.

### Alu-PCR assay

Due to a frequently observed background synthesis in the Repli-G amplified no-template control, we performed an extra quality control on 1 µl (1∶50 dilution) of unpurified phi29-amplified DNA. An *Alu* specific band should be present in all Repli-G amplified samples and absent in the Repli-G amplified no-template control. Each 2 µl of sample was compared to 1 µl serial dilutions of male control DNA (Promega, WI, USA) (10, 5, 2, 1, 0.5, 0.1, 0.01, 0.001 ng/µl). The 25 µl reactions contained 1× Hot start SYBR green master mix (Qiagen), 1.5 mM MgCl_2_, 0.2 mM dNTPs Mix, 400 nM primers each (*Alu*-forw: 5′-GTGGGCTGAAAAGCTCCCGATTAT-3′ and *Alu*-rev: 5′-ATTCAAAGGGTATCTGGGC TCTGG-3′). The cycling conditions were as follows: 1 min at 94°C; 35 cycles of 20 s at 94°C, 20 s at 55°C and 20 s at 72°C; 10 min at 72°C. The amplification products were checked by melting curve analysis using the LightCycler™ quantification software 3.5 (Roche Diagnostics, Mannheim, Germany) and gel electrophoresis. Amplification was considered successful when the amount of human *Alu* sequences in phi29 generated DNA fragments ranged from 10–30% and when a smear of DNA fragments, ranging from 1 to 20 kb, was visible by gel electrophoresis.

### ArrayCGH

10 µg of amplified and purified DNA were labeled using the BioPrime Array CGH Genomic Labeling kit (Invitrogen) according to the manufacturer's instructions in a volume of 50 µl with a modified dNTP mix containing 120 µM each of dATP, dGTP, and dCTP; 60 µM dTTP; and 60 µM Cy5-dUTP or Cy3-dUTP. Labeled DNA was purified using QIAquick PCR purification columns (Qiagen). Tumor and reference DNA were pooled, mixed with 50 µg Cot-1 DNA (Invitrogen) and concentrated to a volume of 20 µl in a vacuum centrifuge. DNA was then mixed with an equal amount of 2× formamide buffer, denaturated at 95°C for 5 min and preincubated at 37°C for 30 min in a waterbath.

Hybridizations were carried out on custom (printed at the NHGRI/NIH Microarray Core Facility) glass slides containing the Operon 70mer oligonucleotide set version 3 with 34.580 oligonucleotides. The oligonucleotides were originally designed for expression analysis. For arrayCGH all oligonucleotides were sequence aligned against the human genome (build 34), EnsEMBL, RefSeq, dbEST and UCSC known genes resulting in 29.383 oligonucleotides with a specific chromosomal mapping.

DNA samples were hybridized onto the array for 16–18 hours at 42°C utilizing the MAUI 4-bay hybridization system and MAUI Mixer A0 (BioMicro System, Salt Lake City, UT, USA). After hybridization arrays and MAUI Mixer were disassembled in 42°C 1×SSC and 0.05% SDS (wash solution 1) and subsequently washed two times in wash solution 1 for 5 minutes followed by two washes in 0.1×SSC (wash solution 2) for 5 minutes. Dried array slides were scanned using a ScanLite Express microarray scanner (Perkin Elmer, Wellesley, MA, USA). Raw image files of the arrays were processed using DeArray software [23] and resulting image data were imported into R environment using bioconductor packages [24] for further analysis.

### Data processing and statistical analysis of microarray data

The statistical analysis for identifying chromosomal regions with altered copy numbers in single arrays consisted of two steps. First, after mapping the measured gene copy number log-ratios to their respective chromosome locations, smoothing was applied. The adaptive weights smoothing-based algorithm GLAD [25] for identifying regions of constant copy number was used. This algorithm fits a piecewise constant function along the chromosome to the log-ratios. Then, for every sample regions that are amplified or deleted were identified. Here, normal distributions were fitted in a robust way to the smoothed log-ratios of each array. In particular, the median and the interquartile range were calculated, and a normal distribution was fitted to these parameters. Finally, constant regions with values above or below specified cutoffs were detected as gained or lost regions, respectively. In order to discriminate between weak and strong signals, the median plus or minus one or two standard deviations were selected as cutoffs for weak and strong aberrations, respectively. The algorithms were implemented in the R programming language (www.r-project.org). The results were obtained using R version 2.3 and the GLAD library provided by the Bioconductor project (www.bioconductor.org), version 1.7.

### Chromosomal CGH

Hybridization was done as described previously with minor modifications [26]. 500 ng biotin labeled probe DNA, 500 ng digoxigenin labeled reference DNA (human male reference DNA, Promega, WI, USA) and 50 µg human unlabeled cot-DNA (Invitrogen, Karlsruhe, Germany) were precipitated with 0.3 M sodium acetate in the 2.5 fold volume of ethanol and dissolved in 2.5 µl deionized formamide. After 30 minutes, the 2.5 µl of the double hybridization mix (100 mM sodium phosphate buffer, 4×SSC and 20% dextrane sulfate, pH 7.0) were added and denatured for 5 min at 75°C followed by 15 min preannealing at 37°C. The hybridization was performed under sealed coverslips for 3 days at 37°C in a moist chamber. After hybridization, the slides were washed three times for 5 min in a washing solution (50% formamide in 2×SSC pH 7.0) at 45°C, twice in 2×SSC (pH 7.0) at 45°C, and once in 0.1×SSC (pH 7.0) at 45°C.

Biotinylated probe DNA was detected with FITC labeled streptavidin (Vector Laboratories, Burlingame, CA, USA). The digoxigenized control DNA was detected with rhodamine labeled anti-digoxigenin antibodies (Roche Diagnostics, Mannheim, Germany). Finally the slides were counterstained, and an antifade solution applied in the same step (Vectashield with DAPI, Vector Laboratories).

Slides were analyzed using a digital image analysis system (MetaSystems, Altlussheim, Germany), based on an Olympus AX 70 microscope equipped with a cooled CCD camera (Photometrics, Tuscon, AZ, USA). For analyzing differential fluorescence data we used the MetaSystems ISIS 3 software. The three-color images with red, green, and blue were acquired from 15–20 metaphases. Chromosome imbalances were detected on the basis of the fluorescence ratio profile, deviating from the balanced value (FITC:rhodamine = 1). For each chromosome the final ratio values were prepared from mean values of at least ten chromosome homologues from separate metaphase spreads. CGH results were plotted as a series of green to red ratio profiles, and the interpretation of results followed previously described protocols [3].

### Fluorescence in situ hybridization

FISH was performed on nuclei and metaphase spreads of the cell lines CWR22 and CWR22-Rv1. Metaphase spreads of lymphocytes from a healthy donor were used as a control. The BAC clone RP11-165H19 (9p13.3, GenBank accession number AQ382511) was obtained from BACPAC Resource Center Children's Hospital (Oakland Research Institute, Oakland, CA, USA) and cohybridized with a probe specific for the centromere of chromosome 9 (D9Z1; Oncor, Gaithersburg, MD, USA). Bacterial cultures and DNA isolation were done according to the BACPAC Miniprep protocol (http://www.biologia.uniba.it/rmc). *Alu*-PCR products of the BAC were used as probes and were biotinylated using nick translation. Dual color fluorescence *in situ* hybridization and detection of fluorescence signals were done as described previously [6].

### Quantitative real time PCR

Quantitative real time PCR for gene copy number measurement was based on a recently described approach [27]. In the present study, we used predesigned and validated SNP assays (Applied Biosystems, CA, USA) with the AB 7900 system (Applied Biosystems). For the validation of the 9p13.3 amplicon two genes within the amplicon (IL-11RA, AssayID: C__11340987_10 and DCTN3, AssayID: C__25472566_10) and a gene at at 3p24.2 located in a not altered region in prostate cancer (TOP2B, AssayID: C__8063527_10) were analyzed.

In a first step, all SNP assays used in this study were run on a dilution series of normal blood DNA to determine PCR efficiency (E) of each primer set by the formula E = 10^−1/s^, where s represents the absolute value of the slope in a plot of the threshold cycle (C_T_) against log of input amount. All primers were shown to have an equal efficiency of approximately E = 2 and in relative efficiency plots comparing the different primers (log of input amount *vs.* ΔC_T_) the absolute value of the slope was less than 0.1. Relative gene copy number was then calculated following the equitation: 2^−ΔΔCT^ (User bulletin#2, Applied Biosystems). For calibration normal blood DNA was added in each PCR run. For genes having both alleles detected by the assay the C_T_ value was subtracted by 1 based on the shown efficiency of 2. To determine whether results obtained by real time PCR analysis of the prostate cancer samples were significantly different from those obtained for samples from healthy individuals, we determined a tolerance interval (TI) for the relative gene copy numbers, using the standard deviation (SD) of C_T_ values for target and reference genes in 8 healthy individuals according to the equation: TI = 2±(SDΔC_T_×2). The TI ranged from 1.62 to 3.17 for *DCTN3* and from 1.77 to 2.94 for *IL11-RA*.

## Results

### Minimal recurrent regions of alterations

**Figure 1:**
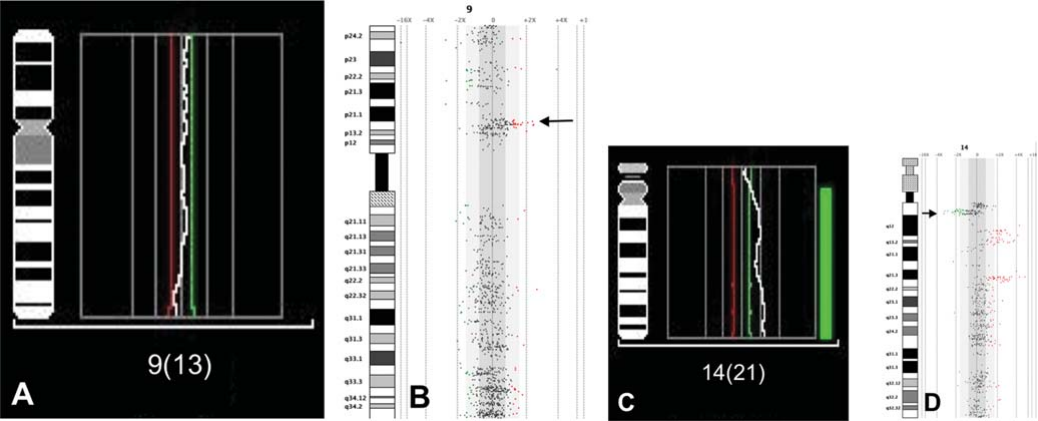
Comparison of cCGH (A, C) and arrayCGH (B, D) results from chromosome 9 of CWR22 and chromosome 14 of DU145-MN1. A small region of gain on 9p close to the centromer (B, arrowhead) and a small deletion on 14q (D, arrowhead) are only detected by arrayCGH.

Because of their highly altered genomes and the large volume of data generated by array analyses, simple visual inspection of altered loci proved inefficient to identify minimal recurrent regions of aberration. To analyze the data set, we combined automatic aberration identification with a modified frequency plot procedure. Application of weighted frequency analysis to the autosomes of prostate cancer cell lines revealed multiple regions of highly recurrent changes. In comparison to our cCGH analyses several small amplifications and deletion units were only detected by aCGH, especially in pericentromeric regions and regions near the telomeres (Fig. 1). The size of the smallest regions of aberration could be determined to 110 kb for losses at 19q13.41 in cell lines DU145*_NIH_*, DU145-MN1, PC3 125-1L and CWR22-Rv1, and 760 kb for gains at 14q32.11-q32.12 in PC3*_HOM_*, PC3-N and PC3-125-1L. Most chromosomal aberrations as detected by cCGH were also seen in aCGH. However, more losses were detected with microarrays and more large regions of gains with metaphase technique. For the commonly detected regions of aberrations, no discrepancy between the two methods in assigning gains or losses to the individual region was observed (table 1).

**Table 1:** Overview of detected chromosomal aberrations by cCGH and aCGH

	Chromosomal aberrations		
Cell line	no. found with both techniques	found only in arrayCGH	found only in cCGH
PC3*_HOM_*	26	-1q24.3, -1q32.2, -3q25.1, -3q27.1-q28, +5q11-5q12.2, +12p12.1-p13, -12p11.2, -12q24.33,	+2p23-p25, +7, +5p14-p15, +11q14-q25
PC3*_NIH_*	16	-1q21-q24.3, +1q25.1-q31.3, +1q41, +5q11-q13.1, -5q32-q33, -17q21.3-q25, +17q21.1	+2p23-p25, +3p, +7
PC3-N	28	-1q21q24.3, +1q25.1-q32.1, +1q43, +17q11, -17q21.31-q25,	+2q21-q24, +7p11-p22, +9q22-q34
PC3-125-1L	17	-1q24.3, +1q21.1-q31.3, +1q43, +Xp21.3-p22	+5q11-q13.1, +7, +11q14-q25, 12q21-q24, +17q22-q25
Du145*_Hom_*	12	+9p13.2-p13.3, +9p21-q34, +12p11.1-p13	+2p14-p25, +7p11.2-p22, +10q22-q26, +11q11-q25, +12q11.2-p24.3, +15, +16q21-q24
DU145*_NIH_*	16	+9p13.2-p13.3, -11p11p15, -17p13.2	+2p14-p25, +10q22-q26, +11q11-q25, +12q11.2-p24.3, +15, +16q21-q24
Du145-MN1	15	+1p12, +11p15.4, 12p12.1, -14q21.3, -17q12, -19q13.4	+2p14-p25, +10q22-q26, +11q13-q14, +15, +16q21-q24
LNCaP	8	-1p33, -11q21.1-q34, -13, -19q13.2-q13.43	-19p13.3
LNCaP-CN4-2	13	-11q12.1, -19q13.2-q13.33	+1q21-q25, +3q24-q26, +5, +9p
CWR22	14	+9p13.3	+8p21-p23, +10q25-q26
CWR22-RV1	20	-2p, -9p21-p24	

### Cytogenetic constitution of different branches of prostate cancer cell lines and parental cell line vs. subline comparison

**Figure 2:**
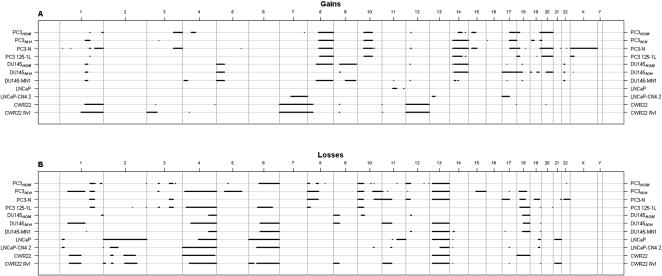
Whole genome plot of gained (A) and lost (B) chromosomal regions of 11 prostate cancer cell lines, as detected with aCGH. Numbers above the plot indicate chromosome numbers and vertical lines boundaries between chromosomes.

The aCGH results of the parental cell lines PC3*_HOM_*, DU145*_HOM_*, LNCaP and CWR22 and their sublines are summarized in figure 2. For PC3 and DU145, two different branches could be compared (*HOM vs. NIH*). Most amazingly, the cytogenetic constitution of PC3*_HOM_* differed markedly from PC3*_NIH_*. From a total of 54 aberrations in both cell lines, only six strong aberrations could be found in common: losses at 1q, 8p, 10p and 13 and gains at 10q and 17q. The gain of 8q11.2-8q24.3 in PC3*_HOM_* was also observed in PC3*_NIH_* showing a minimal size variation of 100 kb. For DU145*_HOM_* and DU145*_NIH_*, a good overall concordance of the genetic composition was found.

Comparing cell lines PC3*_HOM_*, DU145*_HOM_*, LNCaP and CWR22 with their derived sublines PC3-N, PC3-125-1L, DU145-N, LNCaP-CN4-2 and CWR22-Rv1, respectively, a high congruency of the cytogenetic aberrations between the corresponding cell lines was yielded by aCGH. It is worth mentioning that besides minor variations of the number of extra regions with gains or losses of genetic material differences between parental cell lines and sublines predominantly concerned the extent of corresponding regions with copy number alterations, as for example was observed for the regions with gains at 12q and 15q in PC3*_HOM _vs.* PC3-N and for the regions with losses at 2q, 4q, 6q and 13q21.33 in LNCaP *vs*. LNCaP-CN4-2.

### Validation of the amplified pericentromeric region 9p13.3 using FISH analysis and quantitative real time PCR

**Figure 3:**
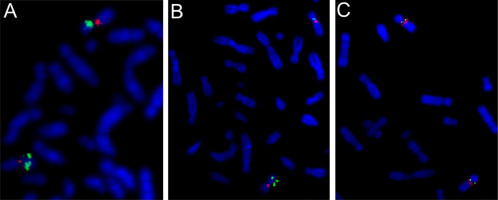
Validation of amplicons by FISH analysis on cell lines. Identification of increased copies of signals of the BAC clone RP11-165H19 mapped to 9p13.3 amplicon in cell line CWR22 and CWR22-Rv1 (A and B). C Optimal signal and lack of cross hybridization was verified using normal metaphase spreads showing two signals for each probe. BAC clone RP11-165H19 signals are green; red signals indicate chromosome 9 centromere (D9Z1).

A novel 1.7 Mb region with copy number gains was consistently recognized in the cell lines CWR22 and CWR22-Rv1 and was mapped to the pericentromeric 9p13.3 subband. The presence of this amplicon in the cell lines CWR22 and CWR22-Rv1 was confirmed by metaphase FISH using a BAC clone (RP11-165H19) from the amplified region and a probe specific for the centromere of chromosome 9 (Fig. 3A and B). Optimal signal and lack of cross hybridization was verified using normal metaphase spreads (Fig. 3C). This BAC clone contained two genes, *IL-11RA* and *DCTN3*, which were already thought to play a role in prostate cancer growth [13].

**Figure 4:**
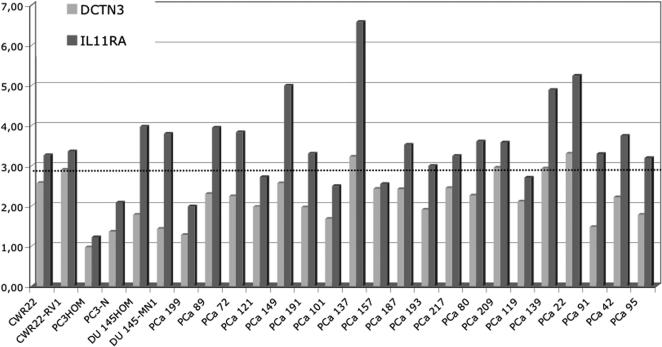
DNA copy number quantification of *DCTN3* and *IL11-RA* genes in prostate cancer cell lines and 20 primary prostate carcinoma samples using quantitative real time PCR. *IL-11RA* showed a significant increase in gene copy number above normal in CWR22, CWR22-Rv1, DU145*HOM* and DU145-MN1 and in 15 out of the 20 (75%) prostate cancer samples. For *DCTN3*, no copy number gain was detected in any cell line and in only 2 out of the 20 (10%) prostate cancer samples. Values above cut off line being assigned as increased gene copy number compared to normal.

Based on gene localization and annotated gene function, we selected these two candidate genes for gene copy number measurements in the prostate cancer cell lines and 20 primary prostate carcinoma samples using quantitative real time PCR. *IL-11RA* showed a significant increase in gene copy number above normal in CWR22 and CWR22-Rv1 and in 15 out of 20 (75%) primary prostate cancer tumor samples (Fig. 4). For *DCTN3*, no copy number gain was detected in any cell line and in only 2 out of the 20 (10%) prostate cancer tumor samples. The control gene *TOP2B* at 3p24.2 was proved unchanged compared to normal blood (data not shown).

## Discussion

Although prostate cancer cell lines have been previously analyzed by aCGH utilizing cDNA microarray platforms [12], [13], [15]–[20], we demonstrate in this study the feasibility of a longmer oligonucleotide set, originally designed for expression analysis, for a high-resolution genomic profiling of nine prostate cancer cell lines. The microarray data for this study have been submitted to NCBI GEO with accession GSE7376. Compared to cCGH, we detected more deletions and small regions of gains with aCGH, especially in pericentromeric regions and regions near the telomeres, where cCGH is known not to deliver reliable informations on DNA imbalances. On the other hand, large regions of gains as revealed by cCGH encompassing almost the whole arm of chromosomes were overlooked by aCGH. Similar observations were also reported by Saramaki et al. (2006) [13] using cDNA-based aCGH. Besides normalization artifacts one might argue that these differences could result from the DNA amplification step for aCGH in our study, whereas non-amplified DNA was used for cCGH. Although whole-genome amplification by phi29 polymerase may add some bias and increase background noise, it is regarded the most unbiased approach compared to PCR-based DNA amplification procedures [28]. As any DNA amplification step seems inevitable in the study of primary prostate cancer samples, we used an amplification step in our study even though DNA quantity was not a limiting factor when working with cell lines.

Our aCGH findings are in good agreement with the data reported in the literature for PC3, DU145, LNCaP, and CWR22 [12], [13], [15]–[20]. However, an interesting side aspect of our study is the observation of a marked cytogenetic difference between the two PC3 branches, which were held in two different labs (PC3*_HOM_ vs.* PC3*_NIH_*). The presumed genomic instability of cell lines may lead to genetic divergency, what has to be considered when comparing findings from different labs on seemingly the same cell lines. A quality management system, which includes details of the genetic composition of the individually used cell lines, seems prudent.

Concerning the comparison of parental cell lines and sublines, no gross differences in the cytogenetic composition were found by aCGH. Differences predominantly concerned the boundaries of corresponding regions with copy number alterations. These findings are in contrast to our previous cCGH studies demonstrating several extra regions of DNA copy number alterations in the sublines compared to the parental cell lines [4]. An explanation might be technical, as the previously reported differences mainly involved large chromosome regions, which are detected with a lower sensitivity by aCGH compared to cCGH, as was discussed above.

As a validation of the high resolution of aCGH, we further analyzed a small amplification unit of 1.7 MB in the pericentromeric region of chromosome 9 at 9p13.3, which was found in the xenograft-derived cell lines CWR22 and CWR22-Rv1 by aCGH but not by cCGH. Interestingly, Saramaki et al. (2006) [13] also found this region by aCGH to be amplified in the prostate cancer xenograft LuCaP35. They confirmed their results by BAC-FISH and demonstrated amplification and overexpression of the ubiquitin-conjugating enzyme gene E2R2 (*UBE2R2*), the dynactin 3 gene (*DCTN3*) and the WD repeat domain 40A gene (*WDR40A*) at 9p13.3. Using a real time PCR technique, we further quantified the copy number of the interleukin 11 receptor alpha gene (*IL-11RA*) and the dynactin 3 gene (*DCTN3*) both revealing the highest log2 ratio in aCGH within 9p13.3. *IL-11RA* was found with the highest copy number gain in CWR22, CWR22-Rv1, DU145*_HOM_* and DU145MN1 suggesting *IL-11RA* as the putative target gene of this amplicon. We, therefore, screened 20 primary prostate cancer samples and found 75% of the tumors harboring an *IL-11RA* copy number gain alone, none of *DCTN3* alone, and 10% of both genes. Mean copy number gain for *IL-11RA* was approximately 4-fold. These data emphasize our initial assumption that *IL-11RA* represents the amplification target rather than *DCTN3*. This hypothesis is further strengthened by immunohistochemical studies [29], [30] and the cancer profiling database Oncomine™ (www.oncomine.org) which both reveal high overexpression of *IL-11RA* in prostate cancer compared to normal prostate tissue. *IL-11RA* encodes a specific receptor for IL-11 and belongs to the family of gp130-dependent cytokine receptors, which include receptors for IL-6, leukemia inhibitory factor, ciliary neurotrophic factor, oncostatin M, and cardiotrophin [31]. An important signaling system activated by *IL-11RA* and other members of this receptor family is the Janus kinase-signal transducer and activator of transcription (Jak-STAT) pathway with STAT3 having a well studied importance in prostate carcinogenesis [32]. Moreover, activated STAT3 is believed to play a key role in androgen receptor activation in the absence of androgens, one explanation of hormone refractory growth of prostate cancer [33]. Whether *IL-11RA* is causative in prostate cancer growth needs to be investigated in further studies.
